# Perfusion Index and Its Correlation With Intraoperative Hypotension in Lower-Segment Cesarean Section Under Spinal Anesthesia: A Prospective Observational Study in a Tertiary Care Hospital in Eastern India

**DOI:** 10.7759/cureus.30431

**Published:** 2022-10-18

**Authors:** Rachana Inamanamelluri, Saswati Das, Laxman K Senapati, Amit Pradhan

**Affiliations:** 1 Anesthesiology, Mamata Medical College, Khammam, IND; 2 Anaesthesiology, Kalinga Institute of Medical Sciences, Kalinga Institute of Industrial Technology Deemed to be University, Bhubaneswar, IND

**Keywords:** lower-segment cesarean section, hypotension, spinal anesthesia, vasopressors, sympathectomy, systemic vascular resistance, subarachnoid block, pulsatile flow, peripheral vasodilation, perfusion index

## Abstract

Background

Hypotension is commonly encountered in patients undergoing lower-segment cesarean section (LSCS) under the subarachnoid block (SAB) owing to decreased vascular resistance caused by the sympathetic blockade and decreased cardiac output because of blood pooling in blocked areas of the body. Perfusion index (PI) is a good indicator of systemic vascular resistance and can foretell hypotension. This study aimed to associate baseline PI with intraoperative hypotension after SAB in LSCS.

Methodology

This was a prospective observational study with a sample size of 50. The baseline PI was recorded every 10 seconds for one minute in a supine position on the right index finger at room temperature of 26°C to 28°C. The blood pressure (BP) and heart rate (HR) were recorded at an interval of one minute for three minutes. The mean of PI, BP, and HR were taken as the preoperative value. Spinal anesthesia was administered as per institutional protocol. Hypotension, defined as mean arterial pressure (MAP) <20% of baseline or MAP <60 mmHg was treated with vasopressors. Regression analysis with the Spearman correlation coefficient was done to correlate PI and hypotension.

Results

The incidence of hypotension in parturients with PI <2.85 was 28.6% (5/20) and in parturients with PI >2.85 was 82.8% (p < 0.001). The requirement of sympathomimetics was higher in parturients with PI >2.85.The area under the receiver operating characteristic curve was 0.8883. A cut-off PI value of 2.85 can identify parturients at risk for central neuraxial block-induced hypotension with a sensitivity of 80% and a specificity of 75% (p < 0.001).

Conclusions

The PI is a useful tool for predicting hypotension in healthy parturients undergoing elective cesarean section under SAB.

## Introduction

Cesarean delivery is the most commonly performed surgical procedure in the obstetric population. It is performed to improve maternal and fetal outcomes or to reduce anticipated complications from spontaneous labor and vaginal delivery. Ease of administration, faster onset of action, and the ability to provide adequate surgical anesthesia make the subarachnoid block (SAB) the anesthetic technique of choice for elective cesarean delivery [1]. In addition, it has the added advantage of postoperative analgesia along with maternal and fetal safety [1]. Pharmacological sympathectomy owing to intrathecal anesthesia leads to peripheral vasodilatation and venous pooling of blood which precipitates hypotension [2].

Gravid uterus with subsequent aortocaval compression causing lower mean arterial pressure (MAP) further aggravates these effects [3]. The heightened sensitivity of nerve fibers to local anesthetics along with decreased responsiveness to vasopressors in pregnancy contribute to severe hypotension following spinal anesthesia for the lower-segment cesarean section (LSCS) [4]. The incidence of hypotension following spinal anesthesia in a normal population is 15-33% [5], whereas in parturients it increases to 60-80% [6]. The degree of hypotension depends on the preoperative vascular tone, compensatory sympathetic activity, and hydration status of the patient.

Severe hypotension increases the risk of fetal complications such as fetal acidosis and bradycardia. Maternal hypotension of a few minutes duration can cause fetal bradycardia even during vaginal delivery. The duration rather than the degree of hypotension is more important in affecting neonatal outcomes [7-9]. Therefore, it is imperative to anticipate, prevent, and treat post-spinal hypotension in parturients as maternal blood pressure directly influences the placental blood flow.

The usual method to measure blood pressure during cesarean section is non-invasive blood pressure (NIBP) monitoring. However, its usefulness is undermined by its failure to detect hypotensive episodes promptly. Perfusion index (PI) is defined as the ratio of pulsatile blood flow to non-pulsatile blood flow in a patient’s peripheral tissue, such as in a fingertip, toe, or ear lobe. It is measured using a pulse oximeter depending on the differential absorption of infrared light [10]. PI can be tailored to evaluate peripheral perfusion dynamics due to changes in peripheral vascular tone [11-13]. Decreased vascular tone corresponds to a higher PI [12]. The peripheral pooling of blood will be more if the vascular tone is less. SAB causes peripheral pooling of blood which leads to reduced venous return and decreased cardiac output and blood pressure. Hence, the higher the PI, the higher the chance of hypotension.

A definitive monitoring method is lacking to speculate on post-spinal hypotension in parturients planned for cesarean delivery. There is a need for an assessment method that is quick, easy to perform, and interpreted easily along with a great degree of sensitivity and specificity. Although a study on the western population [14] concluded that a PI of 3.5 can predict intraoperative hypotension, no widely accepted cut-off value of PI for parturients is available for the Asian population. The cut-off PI value may vary in different ethnic groups necessitating a study in our patient population [13]. Few studies have demonstrated baseline heart rate (HR), heart rate variability, supine stress test, point-of-care ultrasound, cerebral near-infrared spectroscopy, and pleth variability index (PVI) as useful predictors of post-spinal hypotension in parturients [14-18]. However, much of the evidence had shortcomings because of small sample sizes and a lack of consensus on the definition of hypotension. Therefore, the present study was undertaken with the primary objective to establish a correlation between preoperative PI and intraoperative hypotension following spinal anesthesia in LSCS. The secondary objective of the study was to ascertain the requirement of vasopressor concerning PI value.

## Materials and methods

Study participants and study design

This prospective observational study was conducted after approval from the Institutional Ethics Committee (KIIT/KIMS/IEC/133/2019) in the Department of Anaesthesiology, Kalinga Institute of Medical Sciences, Odisha, India from September 2019 to September 2021 with the Clinical Trial Registry (CTRI) number CTRI/2019/10/021784.

American Society of Anesthesiologists (ASA) II patients who were planned for elective LSCS were included in the study after obtaining written informed consent. Patients with placenta previa, pre-eclampsia, gestational diabetes mellitus, body mass index (BMI) >40 kg/m^2^, cardiovascular or cerebrovascular disease, hypothyroidism, hyperthyroidism, hypertension, with baseline MAP <75 mmHg, and those posted for emergency LSCS were excluded from the study.

Anesthetic technique

**Figure 1:**
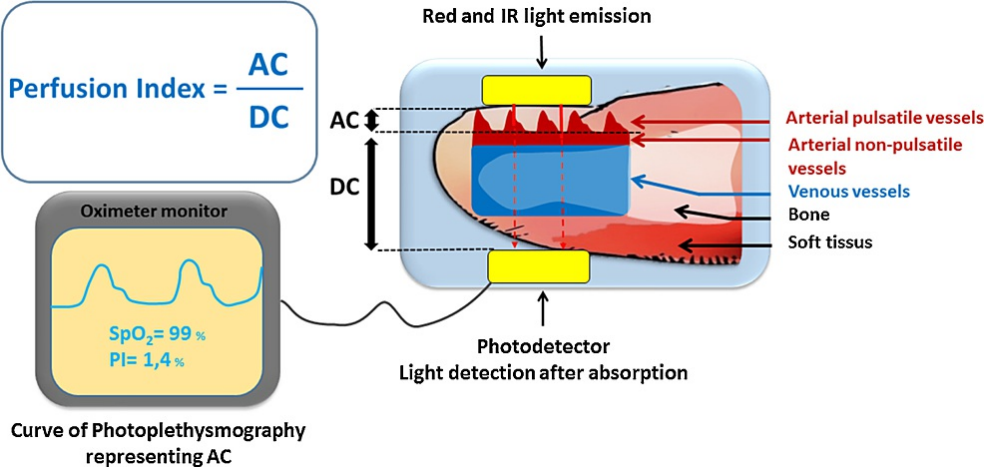
Principles of photoplethysmography and PI calculation. AC: alternating current; DC: direct current; IR: infrared light; PI: perfusion index; PPG: photoplethysmography

**Figure 2:**
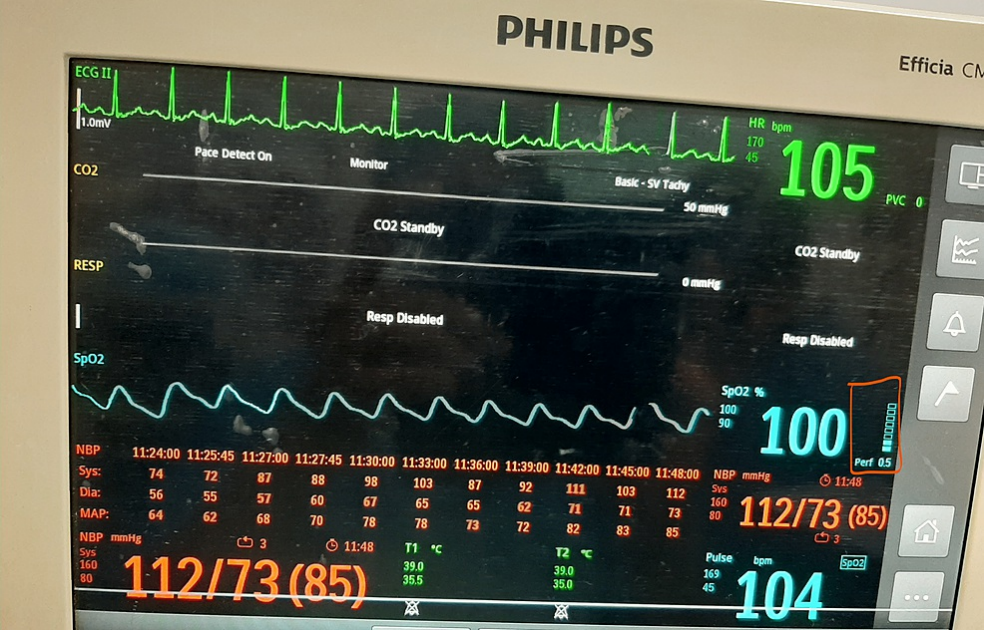
Photoplethysmographic waveform of a parturient with a perfusion index of 0.5.

**Figure 3:**
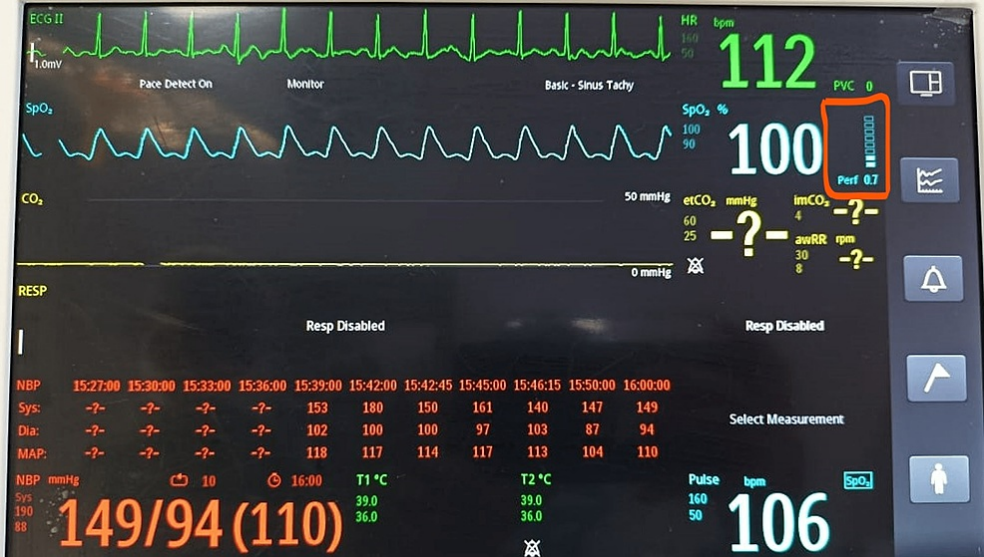
Photoplethysmographic waveform of a parturient with a perfusion index of 0.7.

The fluid deficit was calculated according to the 4-2-1 rule (4 mL/kg/hour for the first 10 kg of weight, 2 mL/kg/hour for the next 10 kg, and 1 mL/kg/hour for each kilogram thereafter). The deficit was corrected over three hours before surgery. In the preoperative room, the baseline PI was monitored with the help of Philips Monitor Efficia CM12 by an anesthesiologist who did not participate further in the monitoring of the patient intraoperatively. The PI was recorded every 10 seconds for one minute in a supine position on the right index finger at room temperature of 26°C to 28°C. PI represents the ratio of pulsatile light absorption to continuous light absorption, i.e., the ratio AC/DC [19] (Figure 1). AC corresponds to the variation of red and infrared light absorption related to the variation of diameters of pulsatile vessels (i.e., arrowed arterial pulsatile vessels in Figure 1). DC corresponds to the light absorption of arterial non-pulsatile vessels, venous vessels, bone, and soft tissues. Some of the plethysmographic waveforms of parturients that we recorded in the preoperative area are depicted in Figure 2 and Figure 3.

The NIBP cuff was always put on the opposite arm. The blood pressure (BP) and HR were recorded at an interval of one minute for three minutes. The mean of PI, BP, and HR were taken as the preoperative value. The patients were transferred to the operation theater and standard monitors were attached, such as electrocardiogram, NIBP, and pulse oximetry. Spinal anesthesia was administered by an anesthetist ignorant of the baseline PI values using Quicke’s 25-gauge needle with 10 mg of 0.5% hyperbaric bupivacaine at L3-L4 interspace in the sitting position. The sensory block level was maintained between T6 and T7 by adjusting the table position. The parturients were excluded from the study if the T6 sensory block level was not obtained and were managed as per institutional protocol. After 20 minutes of spinal anesthesia, maximum cephalad spread was monitored. The PI, HR, BP, and SpO_2_ were monitored continuously and recorded at three-minute intervals till the end of surgery.

Assessment of hypotension and other side effects

The PI, HR, systolic blood pressure (SBP), diastolic blood pressure (DBP), MAP, and SpO_2_ were monitored continuously and recorded at a three-minute interval. All patients were infused with warm saline at a rate of 10 mL/kg/hour. If the MAP dropped more than 20% of preoperative reading or when the MAP <60 mmHg (whichever was greater), then it was regarded as hypotension [20] and treated with a bolus of 6 mg intravenous (IV) ephedrine hydrochloride and/or IV phenylephrine bolus of 1 µg/kg. Bradycardia was defined as HR of less than 55 beats/minute and managed with 0.6 mg of IV atropine. After the delivery of the baby, an injection of oxytocin 10 units was administered in the 500 mL normal saline bottle over the next 30 minutes. The incidence of side effects such as nausea, vomiting, shivering, or any other neurological complications such as transient neurological symptoms was noted till three days postoperatively and managed as per the institutional protocol.

Statistical analysis

The sample size was calculated considering a correlation coefficient of 0.416 which was obtained from the results by Duggappa et al. [15]. With a significance level of 5%, power of 90%, and to detect the significant difference from zero, the sample size was calculated to be 50 by nmster 2.0. Adding a safety factor for drop-offs, a total of 56 patients were analyzed for the study. Data were collected, tabulated, and coded in an MS Excel sheet, and the statistical analysis was carried out using SPSS version 22 (IBM Corp., Armonk, NY, USA). All qualitative parameters were represented as frequency and percentage whereas continuous parameters were reported as mean ± standard deviation. Student’s t-test was used for continuous variables. The chi-square test was adapted to determine the association between selected rows with columns. P-values less than or equal to 0.05 (p ≤ 0.05) were considered statistically significant.

## Results

**Figure 4:**
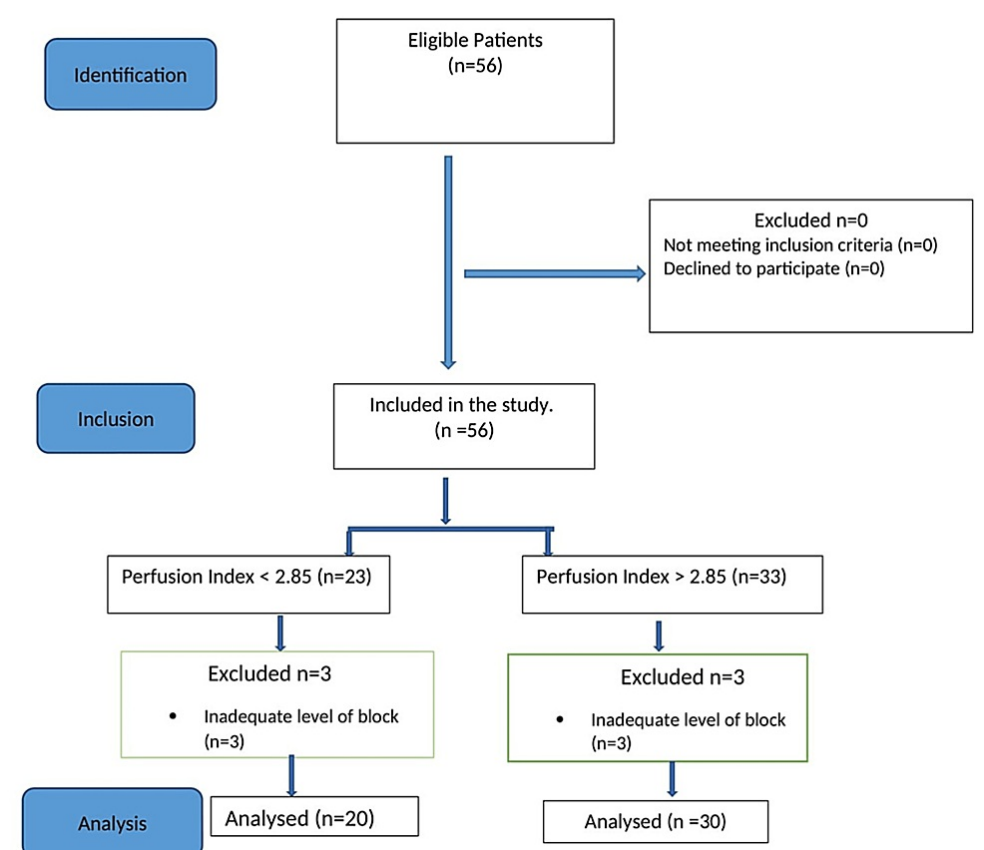
STROBE flow diagram.

A total of 56 patients were included in the study. Because of inadequate levels of the spinal blockade, six parturients were excluded from the study. Among them, four achieved sensory block up to T8 dermatome and two up to T10 dermatome and were managed as per the institutional protocol. Finally, 20 patients fell into the group with a PI value of less than 2.85 and 30 patients into the group with a PI value greater than 2.85 (Figure 4).

Baseline characteristics

A total of 50 patients were included in the study. There was no statistically significant difference (p > 0.05) in age, gestational age, hemoglobin, height, and weight in parturients with PI <2.85 and in parturients with PI >2.85 (Table 1).

**Table 1:** Comparison of age, gestational age, hemoglobin, height, and weight. All data are expressed as mean and standard deviation. PI: perfusion index

	PI <2.85 (n = 20)	PI >2.85 (n = 30)	P-value
Age (years)	30 ± 4.1	29.07 ± 5.049	0.448
Gestational age (weeks)	37 ± 1.37	37.14 ± 1.35	0.913
Hemoglobin (g)	11.95 ± 1.071	11.41 ± 1.18	0.777
Height (cm)	160 ± 9.46	161.86 ± 8.3	0.487
Weight (kg)	66.57 ± 6.638	67.90 ± 8.653	0.560

Incidence of hypotension

**Figure 5:**
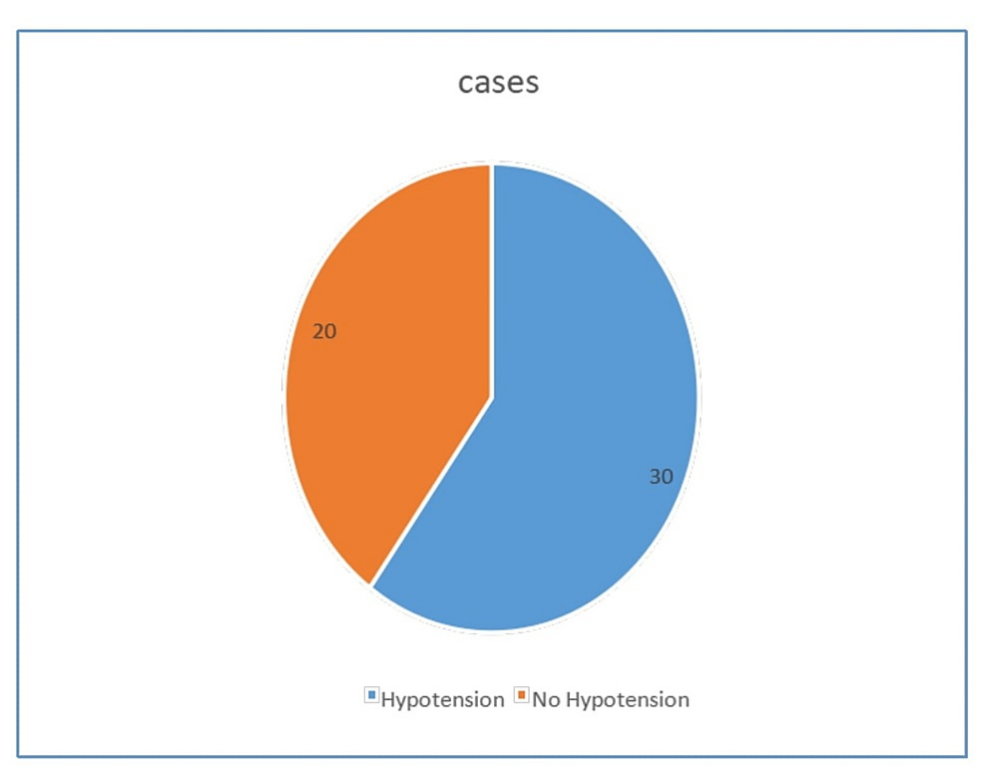
Incidence of hypotension.

In our study, the total number of parturients who had hypotension was 30 and those who did not have hypotension were 20 (Figure 5).

Receiver operating characteristic curve

**Figure 6:**
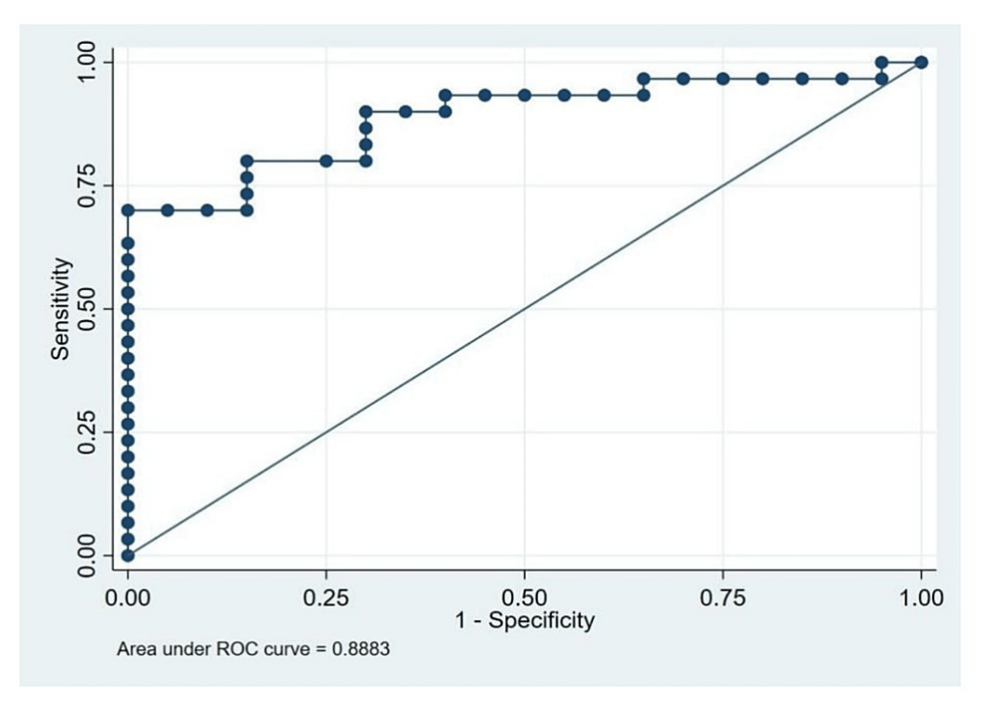
Receiver operating characteristic (ROC) curve depicting baseline perfusion index against the incidence of hypotension.

The ROC curve yielded 2.85 as a more appropriate cut-off with 80% sensitivity and 75% specificity. The area under the ROC curve (AUC) was 0.8883 with a positive predictive value of 82.8 and a negative predictive value of 71.4 (Figure 6, Table 2).

**Table 2:** Characteristics of receiver operating characteristic curve. PPV: positive predictive value; NPV: negative predictive value

Area under the curve	95% confidence interval			
	Sensitivity	Specificity	PPV	NPV
0.883	80%	75%	82.8	71.4

Comparison of hypotension

The incidence of hypotension in parturients with PI <2.85 was 28.6% (5/20) compared to parturients with PI >2.85 was 82.8% (24/30). This was clinically and statistically highly significant (p < 0.001) (Table 3). In the group of parturients having a PI value less than 2.85, all five have only one episode of hypotension. In the group with a PI greater than 2.85, 15 patients had one episode of hypotension, eight patients had two episodes, and one patient had three episodes of hypotension.

**Table 3:** Comparison of hypotension. PI: perfusion index

PI	Hypotension	No hypotension	P-value
<2.85	5	15	<0.001
≥2.85	24	6	

Requirement of vasopressors

The requirement of vasopressors in parturients with PI <2.85 was 28.6% (6/21) whereas in parturients with PI >2.85 was 82.8 (24/29). This was clinically and statistically highly significant (p < 0.001) (Table 4).

**Table 4:** Requirement of vasopressors. PI: perfusion index

PI	Yes	No	P-value
<2.85	5	15	<0.001
>2.85	24	6	

## Discussion

The cardiovascular changes in pregnancy, though physiological, can lead to a decrease in systemic vascular resistance, increased total blood volume, and an increase in cardiac output [15]. The extent of reduction of systemic vascular resistance is influenced by many factors, which, in turn, leads to varying degrees of hypotension in parturients [21-25]. Sympathectomy induced by spinal anesthesia causes a further decline in peripheral vascular tone and increases venous pooling, thereby causing hypotension. A gravid uterus can cause aortocaval compression which coupled with a physiologically decreased vasomotor tone and spinal anesthesia-related sympathetic blockade can further contribute to hypotension in parturients undergoing LSCS under SAB. Hypotension in the mother leads to maternal as well as fetal complications [9]. Thus, the prediction of possible hypotension will aid in preventing the associated complications.

Low BP resulting from reduced systemic vascular resistance leads to a higher PI owing to enhancement in the pulsatile component caused by vasodilatation [12]. Therefore, patients with a higher baseline PI are anticipated to have a reduced vascular tone and are at an enhanced risk of developing hypotension following spinal anesthesia.

PI provides a non-invasive continuous measure of peripheral perfusion. Its utility in predicting peripheral perfusion in different settings was demonstrated by Mowafi et al. [11] for the detection of intravascular injection of the epinephrine-containing epidural test dose, which demonstrated reliability in detecting vasoconstriction. Ginosar et al. [12] demonstrated that an increase in PI following epidural anesthesia was a clear and reliable indicator of sympathectomy. Mehandale et al. [26] concluded in their study that the PI could predict hypotension following propofol induction in patients undergoing general anesthesia with a very high negative predictive value. On the contrary, Yakose et al. reported that PI had no predictive value for hypotension in parturients undergoing LSCS following SAB and concluded that only baseline HR but not other parameters derived from pulse oximetry or heart rate variability were predictors of hypotension associated with spinal anesthesia [27]. This difference could be attributed to different procedural discrepancies, such as the delineation of hypotension, co-loading with colloids, and the process of computing baseline PI.

A value of 3.5 was reported in the studies by Duggappa et al. [15] and Toyama et al. [14] as the cut-off value of the baseline PI to predict hypotension following SAB. Regression analysis and ROC curve analysis inferred that a baseline PI cut-off point of 3.5 could be used to recognize parturients vulnerable to hypotension. Likewise, Malavika et al. [28] reported a cut-off PI of 4.25 with a sensitivity and specificity of 74.5% and 89.7%, respectively, to detect hypotension in parturients with non-severe pre-eclampsia undergoing LSCS. Xu et al. [29] correlated with a high baseline PI of the finger (>3.5) and a low baseline PI of the toe (<2.2) as a predictor of hypotension. However, a study by Jabarulla et al. [30] proposed a PI value of 1.75 which could predict post-spinal hypotension with 75% sensitivity and 71% specificity. The probable reason for getting a cut-off PI value of 2.85 in our study might be because PI can vary in different ethnic groups [13].

The sensitivity and specificity of our study were 80% and 75%, respectively, with a baseline cut-off of >2.85, the sensitivity and specificity in the study by Toyama et al. [14] were 81% and 86%, respectively, and the sensitivity and specificity in the study by Duggappa et al. [15] were 69.84% and 89.29%c, respectively. In our study, the PPV and NPV of baseline PI were 82.8 and 71.4, respectively. The maternal age and body weight did not have any correlation with the occurrence of post-spinal hypotension in parturients of our study. However, the study conducted by Xu et al. [28] showed maternal age, body weight, and BMI in parturients with post-spinal hypotension were higher compared to those who did not have hypotension.

Our study has several limitations. First, the PI values are quite sensitive to any stimulus such as stress, anxiety, or movement that can lead to sympathetic overactivity. Hence, to negate anxiety proper counseling was done and baseline PI values were recorded carefully avoiding any movement. Second, hemodynamic parameters such as cardiac output, systemic vascular resistance, and central venous pressure were not measured as arterial and central lines are not mandatory for uncomplicated elective cesarean delivery. Third, we did not analyze arterial blood gas to exclude hypoxia of the mother and the fetus owing to hypoperfusion.

## Conclusions

The incidence of hypotension was significantly higher in parturients with a baseline PI of more than 2.85 compared to those with a baseline PI of less than 2.85. Hence, the cut-off PI value of 2.85 helps in identifying parturients at enhanced risk of spinal anesthesia-induced hypotension, thereby reducing its deleterious effects by early intervention. Hence, we recommend utilizing PI, a valid tool, as a non-invasive aid to anticipate hypotension during spinal anesthesia for cesarean delivery in day-to-day practice. Future studies are justified to study the minute-to-minute relationship between PI and changes in blood pressure, thereby providing a better correlation between the two variables.
